# 

**Published:** 1920-04

**Authors:** 


### ANNOUNCEMENTS

THE NATIONAL ANAESTHESIA RESEARCH SOCIETY

At a meeting of the Board of Governors of the National Anaesthesia Research Society, held in Cleveland in March, it was voted to have the annual convention of the Society at Pittsburg the week of October 4th, this meeting to be in conjunction with that of the Interstate Anaesthetists' Association and the Pennsylvania Medical Society. It is possible that the Western Pennsylvania Dental Association also will join in the meeting.

In order to augment interest in the primary purpose of the Society, which is research, the governors voted two hundred dollars to be apportioned in prizes for the best papers on research in anaesthesia, such papers to be read at the national meeting. This offer is open to all students, surgical, medical and dental practitioners in the United States.

Canvass of hospitals having revealed need for a uniform anaesthesia chart, a committee of three was appointed to prepare forms. The committee consists of Dr. A. F. Erdman of Brooklyn, Dr. A. H. Miller of Providence, and Dr. E. I. McKesson of Toledo. It was also decided to prepare and publish at the earliest moment possible a monograph on the best practices in anaesthesia in obstetrics.

Announcement was made of the acceptance of the following well-known physicians, dentists and anaesthetists as members of the research committee: Dr. F. C. Mann, Rochester, N. Y.; Dr. John Evans, Buffalo, N. Y.; Dr. A. E. Guedell, Indianapolis, Ind.; Dr. Wm. Harper DeFord, Des Moines, Ia.; Dr. W. E. Burge, University of Illinois; Dr. Wm. Hamilton Long, Louisville, Ky.; Dr. J. Griffith Davis, Baltimore, Md.; Dr. J. J. Buettner, Syracuse, N. Y.; Dr. Tyler, Philadelphia; Dr. Isabella C. Herb, Chicago; Dr. A. F. Erdman, Brooklyn; Dr. A. H. Miller, Providence; Dr.

W. B. Howell, Montreal, Canada; Dr. R. S. Hopkinson, Milwaukee; Dr. Oel E. Lamphear, Kalamazoo; Dr. W. I. Jones, Columbus; Dr. Theo. Casto, Philadelphia; Dr. S. P. Reimann, Philadelphia; Dr. John Polak, Brooklyn, N. Y.

T. T. FRANKENBERG,
16 E. Broad St.,
Columbus, O.

---

### MICHIGAN STATE DENTAL SOCIETY

The next annual meeting of the Michigan State Dental Society will be held at the Hotel Statler, Detroit, Mich., April 12, 13 and 14, 1920.

The chairman on local arrangements is William H. Waller, 613 Washington Arcade, Detroit, Mich., and the secretary, Claude S. Larned, 614 Post Building, Battle Creek.

CLAUDE S. LARNED,
Battle Creek, Mich.

---

### AMERICAN INSTITUTE OF DENTAL TEACHERS

At the annual meeting of the American Institute of Dental Teachers, held at Detroit in January, 1920, the following officers were elected: Dr. Arthur D. Black, Chicago, president; Dr. Guy S. Millberry, San Francisco, vice-president; Dr. Abram Hoffman, Buffalo, secretary-treasurer. Executive Board: Dr. A. H. Hipple, Omaha; Dr. A. E. Webster, Toronto, Ont.; Dr. E. D. Coolidge, Chicago.

ABRAM HOFFMAN, *Sec'y*,
381 Linwood Ave.,
Buffalo, N. Y.

---

### INDIANA STATE DENTAL SOCIETY

The next meeting of the society will be held in the Claypool Hotel of Indianapolis, May 17, 18, 19 and 20. An unusual programme is being prepared.

A. J. KIMM, *Sec'y*.

### Dental Society of the State of New York

The fifty-second annual meeting of the Dental Society of the State of New York will be held in Albany, May 13, 14 and 15, 1920, at the Hotel Ten Eyck. An interesting program of essays and clinics is being prepared and will be announced later. All ethical dentists residing in New York and adjoining states are cordially invited to attend the meeting.

---

### Dental Department of the University of Buffalo

*Alumni Association.* The Alumni Association of the Dental Department of the University of Buffalo will hold its annual meeting at the Hotel Iroquois, on April 15, 16 and 17, 1920.

The meeting will be a part of an alumni post-graduate week. The week of April 12th will afford an opportunity for every dentist who chooses to take post-graduate work from the following men:

Rupert E. Hall, D.D.S., Chicago, Ill. "Full Prosthesis."

W. Earnest Cummer, D.D.S., Toronto, Ont. "Attached Plates and Removable Bridgework."

Clifford E. Rose, D.D.S., Buffalo, N. Y. "Conduction Anaesthesia."

Leuman M. Waugh, D.D.S., New York City. "Radiology."

